# Vertebral HU value and the pectoral muscle index based on chest CT can be used to opportunistically screen for osteoporosis

**DOI:** 10.1186/s13018-024-04825-6

**Published:** 2024-06-07

**Authors:** Xiong-Yi Wang, Sheng Pan, Wei-Feng Liu, Yi-Ke Wang, Si-Min Yun, You-Jia Xu

**Affiliations:** Department of Osteoporosis, The Second Affiliated Hospital of Soochow University, Suzhou, 215000 China

**Keywords:** Osteoporosis (OP), Computed tomography (CT), Pectoral muscle index (PMI), Dual-energy X-ray absorptiometry (DXA), Bone mineral density (BMD)

## Abstract

**Background:**

Existing studies have shown that computed tomography (CT) attenuation and skeletal muscle tissue are strongly associated with osteoporosis; however, few studies have examined whether vertebral HU values and the pectoral muscle index (PMI) measured at the level of the 4th thoracic vertebra (T4) are strongly associated with bone mineral density (BMD). In this study, we demonstrate that vertebral HU values and the PMI based on chest CT can be used to opportunistically screen for osteoporosis and reduce fracture risk through prompt treatment.

**Methods:**

We retrospectively evaluated 1000 patients who underwent chest CT and DXA scans from August 2020–2022. The T4 HU value and PMI were obtained using manual chest CT measurements. The participants were classified into normal, osteopenia, and osteoporosis groups based on the results of dual-energy X-ray (DXA) absorptiometry. We compared the clinical baseline data, T4 HU value, and PMI between the three groups of patients and analyzed the correlation between the T4 HU value, PMI, and BMD to further evaluate the diagnostic efficacy of the T4 HU value and PMI for patients with low BMD and osteoporosis.

**Results:**

The study ultimately enrolled 469 participants. The T4 HU value and PMI had a high screening capacity for both low BMD and osteoporosis. The combined diagnostic model—incorporating sex, age, BMI, T4 HU value, and PMI—demonstrated the best diagnostic efficacy, with areas under the receiver operating characteristic curve (AUC) of 0.887 and 0.892 for identifying low BMD and osteoporosis, respectively.

**Conclusions:**

The measurement of T4 HU value and PMI on chest CT can be used as an opportunistic screening tool for osteoporosis with excellent diagnostic efficacy. This approach allows the early prevention of osteoporotic fractures via the timely screening of individuals at high risk of osteoporosis without requiring additional radiation.

## Introduction

Osteoporosis (OP) is a widespread metabolic bone disease characterized by the loss of bone, resulting in a high risk of fracture [1]. Fractures are one of the most serious consequences of osteoporosis, with high mortality and disability rates, and represent just one of the public health problems that countries must address [2]. However, the diagnostic rate for osteoporosis is considerably lower than its prevalence [3, 4].

Currently, dual-energy X-ray absorptiometry (DXA) is considered the gold standard for measuring bone mineral density (BMD) [5, 6]. However, DXA is expensive, has not been widely used in many countries and is not available readily in many counties [7, 8]. It has been suggested that osteoporosis can be opportunistically screened by computed tomography (CT) [9]. Currently, the HU values of L1 in abdominal CT is used to predict osteoporosis and has been associated with fragility fractures [10]; Studies have proposed a threshold value for predicting osteoporosis using HU values of lumbar spine with good diagnostic ability [11, 12].

Sarcopenia is defined as the loss of skeletal muscle strength and low muscle mass [13]. A growing body of research has shown that sarcopenia is associated with osteoporosis [4, 14]. Some studies have shown that the psoas major muscle index positively correlates with the total volume of skeletal muscle in the body, and that the risk of osteoporosis can be predicted by measuring the skeletal muscle index on CT [10]. Additionally, further studies have shown that the pectoral muscle index (PMI) strongly correlates with BMD [15, 16].

Unlike abdominal CT, chest CT scans are widely used for lung cancer screening, the diagnosis of pneumonia, and the follow-up of lung nodules [17]. Although few studies have been conducted on the thoracic spine, we speculated that the PMI and vertebral HU value on chest CT could be used to opportunistically screen for osteoporosis. The purpose of this retrospective study was to assess the ability to opportunistically screen for osteoporosis by measuring the PMI and vertebral HU value of patients who underwent chest CT for various indications. We hypothesized that this method could opportunistically screen for osteoporosis in the absence of DXA.

## Methods

### Study participants

**Fig. 1:**
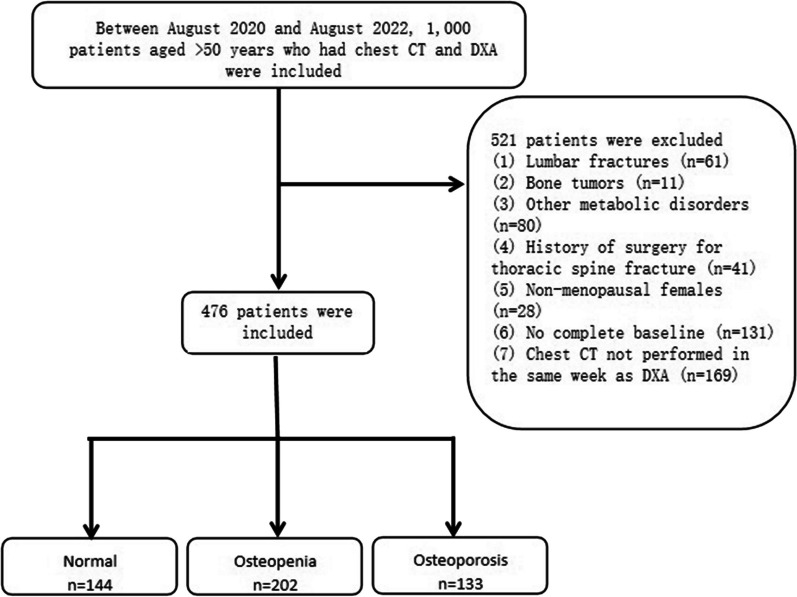
Flowchart of patient inclusion and grouping

The study was approved by committee of the Second Affiliated Hospital of Soochow University and individual consent for this retrospective analysis was waived. In this study, 1000 consecutive participants were enrolled from August 2020–2022. The inclusion criteria were an age ≥ 50 years; having undergone DXA and chest CT. The participants were divided into three groups according to the lowest T-value: osteoporosis (T-score ≤ − 2.5), osteopenia (− 2.5 < T-score ≤ − 1), and normal (T-score > − 1). The exclusion criteria were as follows: combined bone tumor, ankylosing spondylitis, spinal tuberculosis and diffuse idiopathic osteomalacia; a previous history of thoracolumbar spine fracture and surgery; other diseases that may affect bone metabolism [18]; females who were not menopausal; patients with missing baseline data; patients in whom chest CT and DXA were not performed in the same week to minimize time-induced changes in bones and muscles. In total, 479 study participants were included, and baseline data related to age, sex, and body mass index (BMI) were recorded (Fig. 1).

### CT scanning parameters and postprocessing of images

A six-row spiral CT scanner (SOMATOM Emotion 6; Siemens, Munich, Germany) was used for chest CT scanning of the study subjects. The thoracic CT imaging parameters were as follows: tube voltage, 120 kv; tube current, automatic adjustment; and layer thickness, 0.625–2 mm. CT analysis was performed using the Picture Archiving and Communication System (PACS) for medical imaging. In this study, the fourth thoracic vertebra (T4) was used to measure the HU value of the vertebral cancellous bone [19].

**Fig. 2:**
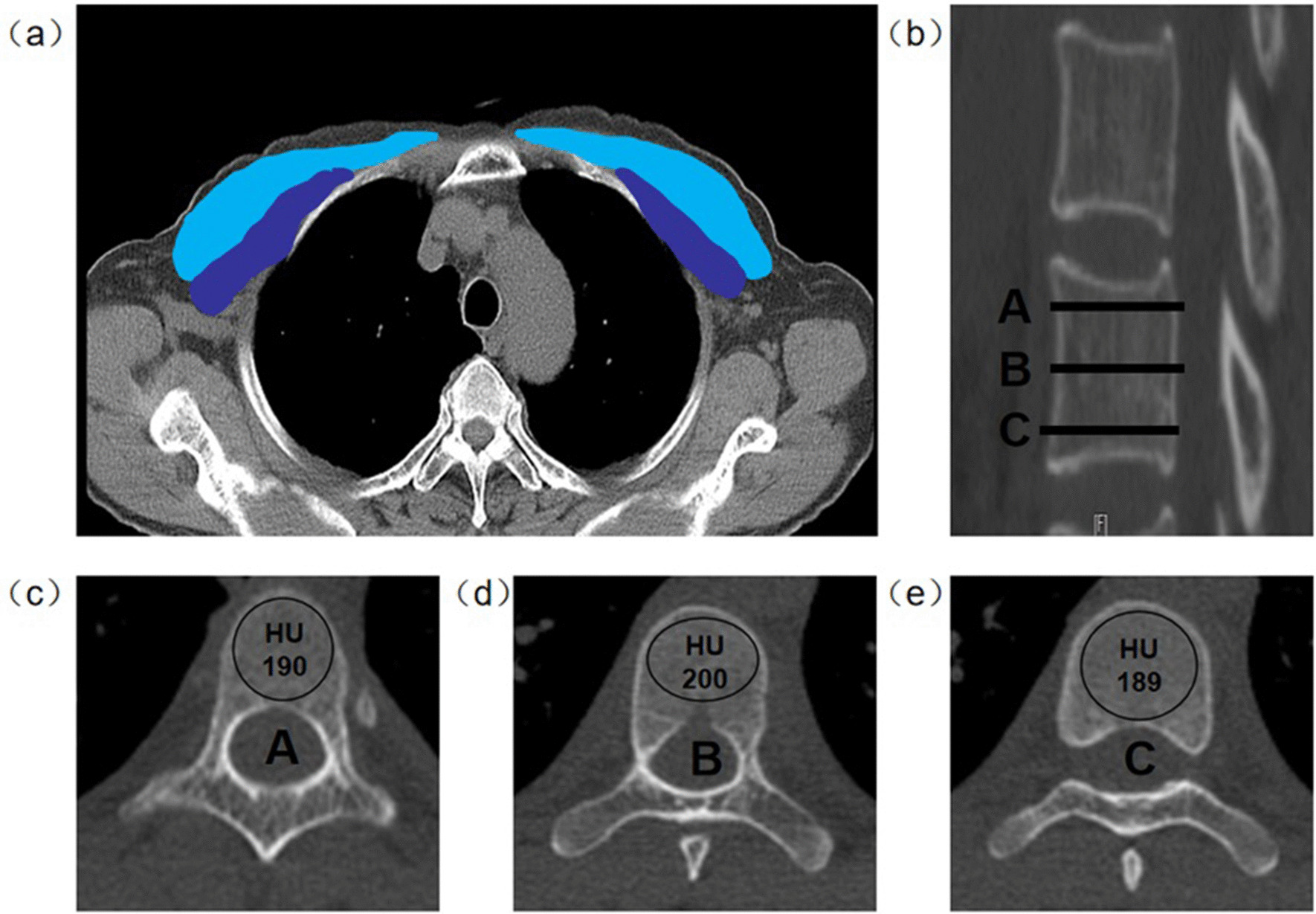
Method for determining the PMA and average vertebral HU values using CT. **a** Horizontal plane of the sternal angle; the pectoralis major is blue, and the pectoralis minor is purple. **b** Vertebral body CT sagittal plane. **c**–**e** CT scan of three levels of a single vertebra in the sagittal plane (A, near the upper end plate; B, middle of the vertebrae; C, near the lower end plate). CT, Computed tomography; PMA, Pectoralis muscle area

A region of interest (ROI) was placed in the cancellous bone region of the vertebral body, covering as much of the cancellous bone region as possible. For each measurement, the ROI did not include the cortical margins, focal lysis, sclerotic lesions, or fractures. Most previous studies only analyzed a single elliptical ROI to evaluate the BMD in each vertebra [20]. Measuring only a single ROI may lead to questionable measurement reliability and reproducibility because of the three-dimensional structure of the vertebrae; therefore, the ROIs in this study were measured separately at three levels on the sagittal images of the target vertebrae: immediately below the upper endplate, in the middle of the vertebrae, and immediately above the lower endplate (Fig. 2).

The average HU values for each vertebra was calculated from the HU value at the three axial levels, with the units expressed in Hounsfield units (HU). At the T4 level, the pectoralis major and pectoralis minor edges were drawn by hand, and the bilateral pectoralis muscle area (PMA) was recorded. Finally, the PMA was corrected for height to obtain the PMI (Fig. 2). The PMI was calculated by dividing the PMA in cm^2^ by the height squared in meters (cm^2^/m^2^) [21]. All measurements were performed by two independent observers, who were unaware of the subject’s DXA measurements, to avoid subjective influences on the measured data [22, 23].

### DXA scanning

The BMD was measured in all study participants using DXA (Lunar Prodigy dual-energy X-ray bone densitometer; GE Healthcare, Chicago, Illinois, USA). Measurements included BMD and T-scores for L1-4 and the hips. The BMD is expressed as g/cm^2^. In this study, the participants were classified according to the lowest T-score of DXA.

### Statistical methods

The observer agreement was compared by calculating the intraclass correlation coefficient (ICC). The interobserver variability of the HU values measurements was determined by a Bland–Altman diagram [24], and consistency was determined by plotting the mean measurement difference of ± 1.96 standard deviation (SD). Normally distributed variables are expressed as the mean ± SD. Differences in continuous variables between multiple groups were determined using ANOVA (for conformity to a normal distribution) and the Kruskal–Wallis test (for conformity to a non-normal distribution); Tukey’s method was used for comparisons between any two groups. Differences in categorical data were tested using the chi-squared test. The Pearson correlation coefficient was used to analyze the correlation between the influencing factors and to plot the matrix. The value of the joint prediction model for opportunistic osteoporosis screening was evaluated using a receiver operating characteristic (ROC) curve; the area under the ROC curve (AUC), sensitivity, specificity, and threshold were calculated. *P*-values < 0.05 was considered statistically significant. Statistical analyses were calculated using SPSS 26.0

## Results

### Clinical baseline data

**Fig. 3:**
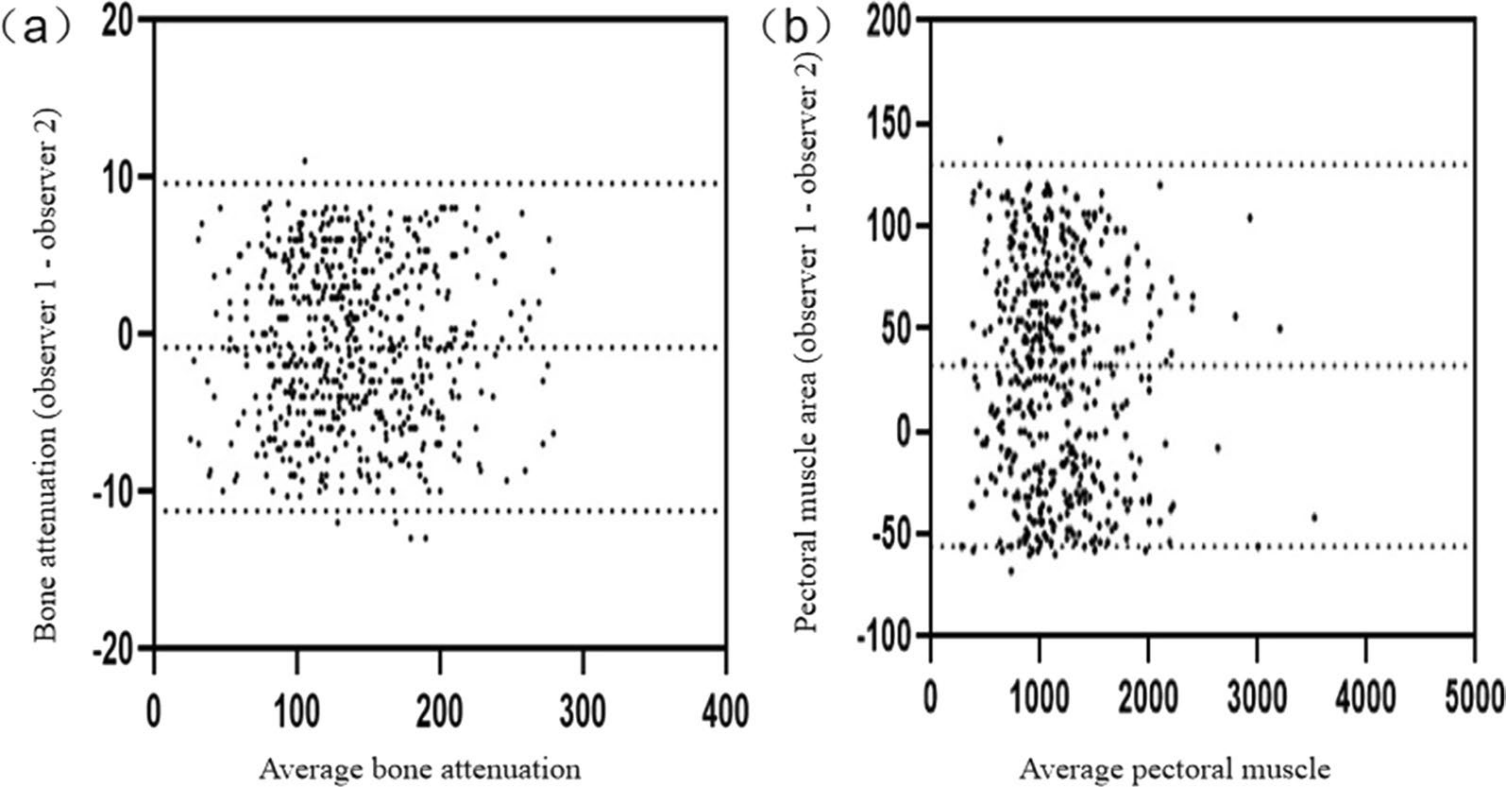
Bland–Altman plots for the interobserver variability on chest CT measurements. **a** Bone attenuation. **b** Pectoral muscle. CT, Computed tomography

One thousand participants were included in this study, among whom 521 were excluded according to the exclusion criteria; finally, a total of 479 participants were enrolled in the study of opportunistic screening for osteoporosis. The measurements of the two observers showed good agreement (ICC_HU_ = 0.994, ICC_PMA_ = 0.992, *p* < 0.05). Figure 3 shows the interobserver variability of the vertebral cancellous bone HU values and PMA measurements; the 95% limits of agreement in the Bland–Altman plot between the two observers ranged from –11.25–9.58 HU for cancellous bone, and − 69.1–132.6 mm^2^ for PMA (Fig. 3).

According to the DXA results, the 479 participants were divided into three groups: normal (n = 144, 30.1%), osteopenia (n = 202, 42.2%), and osteoporosis (n = 133, 27.8%). The data showed that the age, BMI, T4 HU value, and PMI were significantly different between the two groups. In the normal, osteopenia, and osteoporosis groups, the percentage of females and mean age increased sequentially, while the BMI decreased sequentially. In the normal, osteopenia, and osteoporosis groups, the proportions of postmenopausal females were 54.2%, 71.3%, and 81.2%, and the mean ages were 63.8 ± 9.0 years, 68.3 ± 9.1 years, and 74.3 ± 8.9 years, respectively. The mean T4 HU value were 186 ± 42.6 HU, 141 ± 34.4 HU, 106 ± 32.6 HU; the mean PMAs were 29.3 ± 8.2 cm^2^, 23.2 ± 6.7 cm^2^, 17.0 ± 6.1 cm^2^; and the height-corrected PMIs were 11.2 ± 3.0 cm^2^/m^2^, 9.3 ± 2.6 cm^2^/m^2^, 7.1 ± 2.6 cm^2^/m^2^, respectively. Regarding the HU value, PMA, and PMI, two-by-two comparisons revealed that the values for each of these decreased sequentially in the three groups; the differences were all statistically significant (Table 1).

**Table 1:** Comparison of clinical baseline data, HU values, PMA, and PMI in the three groups

Features	Normal (n = 144)	Osteopenia (n = 202)	Osteoporosis (n = 133)	*P*-value
Age	63.8 (9.0)	68.3 (9.1)	74.3 (8.9)	< 0.05
Women	78 (54.2%)	144 (71.3%)	108 (81.2%)	< 0.05
BMI	26.0 (3.0)	24.9 (3.5)	22.4 (3.5)	< 0.05
L1-L4 BMD (g/cm^2^)	1.19 (0.15)	1.0 (0.14)	0.8 (0.13)	< 0.05
Hip BMD (g/cm^2^)	0.95 (0.11)	0.76 (0.08)	0.62 (0.09)	< 0.05
L1-L4 T-score	0.74 (1.32)	− 0.93 (1.21)	− 2.57 (1.10)	< 0.05
Hip T-score	− 0.09 (0.73)	− 1.49 (0.60)	− 2.64 (0.75)	< 0.05
T4 (HU)	186 (42.6)	141 (34.4)	106 (32.6)	< 0.05
PMA (cm^2^)	29.3 (8.2)	23.2 (6.7)	17.0 (6.1)	< 0.05
PMI (cm^2^/ m^2^)	11.2 (3.0)	9.3 (2.6)	7.1 (2.6)	< 0.05

*P*-value: Comparison of any two of the three groups: normal, osteopenia, and osteoporosis

BMI, Body mass index; BMD, Bone mineral density; PMA, Pectoralis muscle area; PMI, Pectoral muscle index

### Correlations between age, BMI, T4 HU value, PMI, and BMD and T-scores

Table 2 shows that age negatively correlated with BMD and T-scores for L1-4 and the hip; a stronger correlation was observed with hip than L1-L4 BMD and T-scores. BMI positively correlated with BMD and T-scores for L1-4 and the hip, with the strongest correlation observed for L1-L4 BMD (r = 0.359, *p* < 0.001).

**Table 2:** Correlation between age, BMI, vertebral HU values, and PMI and T-scores and BMD at each site

	L1-L4 BMD T-score	L1-L4 BMD	Hip BMD T-score	Hip BMD
Age	− 0.183**	− 0.192**	− 0.475**	− 0.451**
BMI	0.343**	0.359**	0.352**	0.324**
T4 HU	0.698**	0.699**	0.642**	0.654**
PMI	0.412**	0.405**	0.441**	0.474**

The correlation was significant at a 0.01 level (two-tailed)

**Fig. 4:**
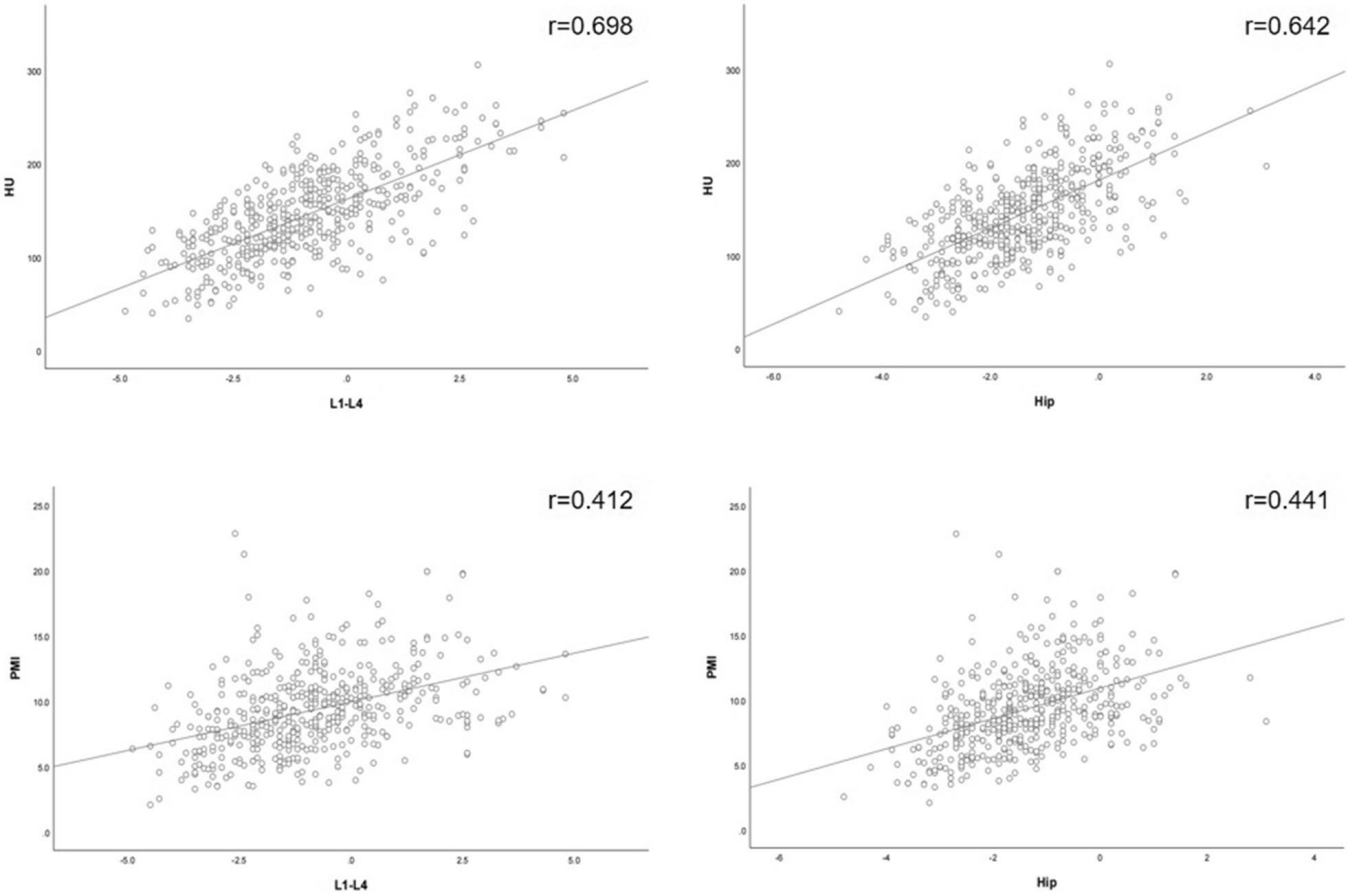
Scatter plots and fitted curves of T4 HU and PMI versus T-scores

The T-scores for L1-4 and the hip strongly correlated with both T4 HU value and PMI, with T4 HU value moderately correlating with L1-4 and hip T-scores (r = 0.698, *p* < 0.01; r = 0.642, *p* < 0.01). Additionally, the correlation with L1-4 and hip T-scores were stronger for T4 HU value than PMI (r = 0.412, *p* < 0.01, r = 0.441, *p* < 0.01). In particular, the correlation between T4 HU value and L1-4T-scores was stronger, whereas that between PMI and L1-4T-scores was weaker than in the hip (Fig. 4).

### Univariate regression analysis of low BMD and osteoporosis

The one-way regression analyses for predicting the risk of low BMD and osteoporosis are summarized in Table 3. Sex, age, BMI, T4 HU values, and PMI were significant predictors of low BMD and osteoporosis. Female sex and age were significant positive predictors of low BMD and osteoporosis, whereas BMI, T4 HU value, and PMI were negative predictors. The diagnosis of low BMD and osteoporosis was associated with lower T4 HU value (OR_low BMD_: 0.964, 95% CI 0.957–0.971, *p* < 0.05; OR _osteoporosis_ = 0.963, 95% CI 0.956–0.971, *p* < 0.05) and lower PMI (OR_low BMD_ = 0.725, 95% CI 0.670–0.786, *p* < 0.05; OR _osteoporosis_ = 0.627, 95% CI 0.564–0.697, *p* < 0.05).

**Table 3:** One-way logistic regression analysis of low BMD and osteoporosis

		OR	95% confidence interval	*p-*value
Low BMD	Female sex	2.569	1.703–3.875	< 0.05
	Age	1.083	1.058–1.109	< 0.05
	BMI	0.843	0.794–0.895	< 0.05
	T4	0.964	0.957–0.971	< 0.05
	PMI	0.725	0.670–0.786	< 0.05
Osteoporosis	Female sex	2.017	1.258–3.232	< 0.05
	Age	1.096	1.069–1.123	< 0.05
	BMI	0.762	0.709–0.819	< 0.05
	T4	0.963	0.956–0.971	< 0.05
	PMI	0.627	0.564–0.697	< 0.05

Low BMD, including osteopenia and osteoporosis; non-osteoporosis, including normal and osteopenia groups

### ROC analysis of diagnostic models

Table 4 shows the efficacy of T4 HU value and PMI for the diagnosis of low BMD and osteoporosis. We found that a threshold of 175 HU was 90% sensitive, a threshold of 126 HU was 90% specific and a balanced threshold of 158 HU for distinguishing patients with low BMD by T4. A threshold of 11.82 cm^2^/ m^2^ was 90% sensitive, a threshold of 8.18 cm^2^/ m^2^ was 90% specific and a balanced threshold of 8.60 cm^2^/ m^2^ for distinguishing patients with low BMD by PMI. A threshold of 148 HU was 90% sensitive, a threshold of 106 HU was 90% specific and a balanced threshold of 131 HU for distinguishing patients with osteoporosis by T4. A threshold of 10.28 cm^2^/ m^2^ was 90% sensitive, a threshold of 6.66 cm^2^/ m^2^ was 90% specific and a balanced threshold of 8.13 cm^2^/ m^2^ for distinguishing patients with osteoporosis by PMI.

**Table 4:** Application of ROC curves to evaluate the diagnostic utility of each model for low BMD and osteoporosis

Group		AUC (95% CI)	Youden’s index	balanced threshold	Sensitivity	Specificity	Threshold for 90% sensitivity	Threshold for 90% specificity
Low BMD	T4	0.850 (0.814–0.880)	0.553	158	0.803	0.75	175	129
	PMI	0.761 (0.720–0.798)	0.4263	8.6	0.579	0.847	11.82	8.18
	Model 1	0.876 (0.843–0.904)	0.5869	0.61	0.851	0.736	0.5159	0.8303
	Model 2	0.887 (0.855–0.914)	0.6058	0.563	0.884	0.722	0.5167	0.8201
Osteoporosis	T4	0.831 (0.794–0.863)	0.4976	131	0.79	0.708	148	106
	PMI	0.800 (0.761–0.834)	0.4952	8.13	0.729	0.766	10.28	6.66
	Model 1	0.876 (0.843–0.904)	0.6034	0.277	0.835	0.769	0.149	0.481
	Model 2	0.892 (0.861–0.919)	0.623	0.27	0.857	0.766	0.168	0.475

Model 1, Combined vertebral HU values and PMI; Model 2, Combined vertebral HU values, PMI, sex, age, and BMI; AUC, Area under the ROC curve; CI, Confidence interval; PMI, Pectoral muscle index

The one-way diagnostic models based on T4 HU value or PMI were statistically significant, with AUCs of 0.850 (95% CI 0.814–0.880, *p* < 0.05) and 0.831 (95% CI 0.794–0.863, *p* < 0.05) for T4, and 0.761 (95% CI 0.720–0.798, *p* < 0.05) and 0.800 (95% CI 0.843–0.904, *p* < 0.05) for PMI for low BMD and osteoporosis, respectively. The AUCs of the model1 were 0.876 (95% CI 0.814–0.880, *p* = 0.05) and 0.876 (95% CI 0.843–0.904, *p* < 0.05) for low BMD and osteoporosis, respectively. When including patient sex, age, and BMI together in the model, the AUCs of the model2 were 0.887 (95% CI 0.855–0.914, *p* = 0.001, sensitivity = 0.884, specificity = 0.722) and 0.892 (95% CI 0.861–0.919, *p* < 0.001, sensitivity = 0.857, specificity = 0.766) for low BMD and osteoporosis, respectively.

**Fig. 5:**
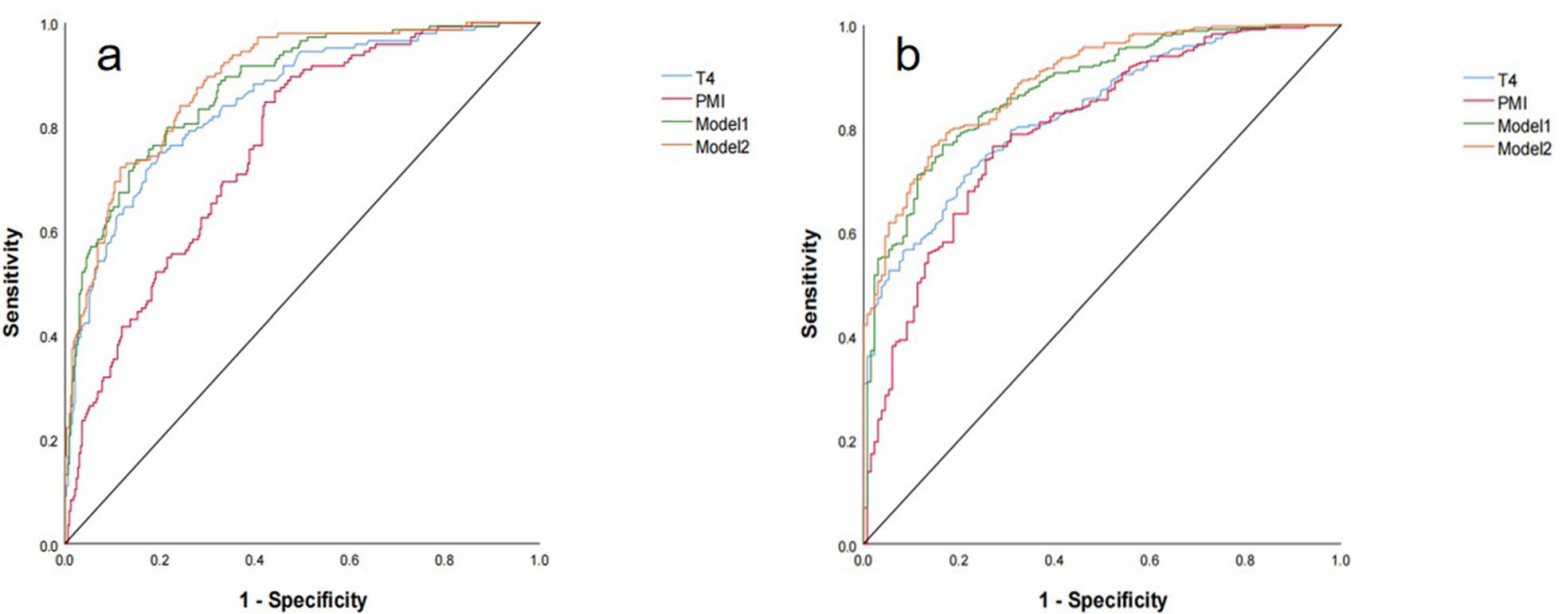
ROC curve of subjects predicting low BMD and osteoporosis. **a** Normal versus low BMD. **b** Non-osteoporosis versus osteoporosis. Low BMD, Including osteopenia and osteoporosis; non-osteoporosis, including normal and osteopenia groups

To better compare the predictive efficacy of the models, ROC plots were plotted (Fig. 5), revealing that the combined diagnostic model (including sex, age, BMI, T4 HU value, and PMI) outperformed the other models.

## Discussion

This study found that low BMD and osteoporosis can be independently screened for by determining the optimal thresholds for T4 HU values and pectoral muscles index during chest CT examinations performed for other reasons. When T4 HU values, PMI, gender, age, and BMI were further included together in the model, it was found that the combined model possessed better diagnostic ability. Our findings are similar to those of previous studies [25, 26]. But unlike previous studies, this is the first time that PMI and T4 HU have been included together in a study, further demonstrating that the combined use of PMI and single vertebral trabecular attenuation values in chest CT examinations is a valuable tool for opportunistic screening for osteoporosis.

CT scans provide rich, high-resolution cross-sectional images and include HU value related to tissue density and cross-sectional area [27] Studies have shown that cancellous bone usually loses BMD faster than cortical bone, bone trabeculae are considered more sensitive indicators of changes in BMD [28]. Therefore, HU values can be used as a basis for detecting reduced bone density [22]. Recent studies have demonstrated that skeletal muscle measurements at the T4 vertebral level on chest CT are as useful as those in the abdomen for assessing whole-body muscle levels; additionally, PMA measurements at the T4 level have been proposed as surrogate markers of sarcopenia [29]. Therefore, the HU value of the T4 cancellous bone and the PMA in the same horizontal plane were used as measurement targets in this study. The T4 HU values and PMA were obtained from measurements taken by two experienced clinicians, with good agreement obtained between the two measurements (ICC_HU_ = 0.994, ICC_PMA_ = 0.992; *p* < 0.05), which is consistent with previous studies demonstrating good inter- and intra-observer reliability for both [30, 31].

As relevant studies have shown that osteoporosis occurs mainly in individuals aged > 50 years [1], our study mainly included patients aged ≥ 50 years. The data demonstrated a gradual increase in the mean age from the normal, to osteopenia, to osteoporosis group; additionally, the proportion of postmenopausal females increased in all three groups. As people age, their body functions gradually deteriorate, including the loss of bone mass; thus, age is a major risk factor for osteoporosis. Postmenopause is another risk factor for osteoporosis, and studies have shown that females aged > 50 years are more likely to develop osteoporosis than males [32, 33]; this may be related to the decline in estrogen levels in females after menopause [34]. Jang et al. [35] demonstrated that HU value were strongly and negatively correlated with age and positively correlated with BMD. Similarly, our findings demonstrated that thoracic spine HU value positively correlated with BMD (r = 0.642–0.698, p < 0.01), and that low thoracic spine HU value are a risk factor for osteoporosis. As bone loss occurs, bone trabeculae become thinner and the BMD decreases, similar to the HU value of the vertebrae. A previous study reported an increased prevalence of osteoporosis in males with COPD and sarcopenia [36]. Our study revealed that PMI positively correlated with the BMD of patients (r = 0.412–0.441, *p* < 0.001). low PMI is a known risk factor for osteoporosis.

In the L1, the optimal cut-off values in the population has been proposed. Pickhardt et al. suggested a threshold of ≤ 110 HU as 90% specific, a threshold of ≤ 160 HU as 90% sensitive and a balanced threshold of ≤ 135 HU for distinguishing osteoporosis from non-osteoporosis [20]. However, there are no authoritative studies that have determined the cut-off values of T4 segments in large populations. Yang et al. had distinguished osteoporosis by a single thoracic spine HU value with AUC values of 0.772–0.834. In T4 segments, the balanced thresholds for distinguishing normal, osteopenia, and osteoporosis were 181 HU, 158 HU, and 131 HU, respectively [26]. This is in contrast to our study, which found that T4HU had good discriminatory ability in distinguishing between low BMD and osteoporosis patients with AUC values of 0.850 and 0.831, respectively. However, the cut-off values in this study were 175 HU and 148 HU, respectively, which is attributed to the difference in subject subgroups, osteopenia and osteoporosis were combined as low BMD in this study. Ronnie Sebro et al. noted an optimal cutoff value of 192 for T4 and an AUC value of 0.76 for diagnosis of low BMD which has a higher threshold and lower AUC value than the present study [37]. However, there is previous evidence of inter-study variation, which highlights likely variable performance of CT for osteoporosis screening across various settings and populations [38]. This may be attributable in part to differences in scanning equipment and protocols [39]. Nevertheless, our findings are consistent with many other studies that T4 attenuation values in chest CT can differentiate osteoporosis.

Mechanical and endocrine correlations have been found to exist between the skeletal muscle and bone; mechanical loading is the most direct link and the key mechanism linking these two tissues to their central role in physical activity. Skeletal muscles also secrete various cytokines, including interleukin (IL)-6, basic fibroblast growth factor (FGF-2), insulin-like growth factor-1 (IGF-1), osteocalcin, and bone-activating hormones, all of which influence the growth and differentiation of osteoblasts and osteoclasts, thus affecting bone function [40, 41]. This observation demonstrates the relationship between skeletal muscle and osteoporosis at the cellular and molecular levels.

Parulekar et al. [15] demonstrated that a low PMI was a positive predictor of osteoporosis in patients awaiting lung transplantation. No study has yet distinguished osteoporosis by determining the optimal cutoff value for PMI. We found that the optimal cutoff values for differentiating low BMD and osteoporosis from the population by PMI were 8.60 cm^2^/ m^2^ and 8.13 cm^2^/ m^2^, with AUCs of 0.761 and 0.800, respectively, and that their diagnostic efficacy was significantly better than that of the lumbar major muscle index in another study (AUC = 0.4). We speculate that this may have been influenced by muscle and sample size. The study by Huang et al. [42] on the psoas major index at the L3 level included a total of 180 patients, which is a much smaller sample size than that in our study.

We further included T4 HU value, PMI, sex, age, and BMI together in the model, and found that the combined model possessed better diagnostic ability than a one-factor model, with AUCs of 0.887 and 0.890, respectively. Moreover, the combined model showed good accuracy and specificity, with sensitivities of 0.884 and 0.857, and specificities of 0.722 and 0.766 for low BMD and osteoporosis, respectively.

In the analysis of CTs obtained for other clinical reasons, although specific imaging software is required, the method described in this study for measuring T4 HU value and PMI requires very little time. At the same time, the method does not require additional equipment, radiation exposure, or patient counselling time. Therefore, the opportunistic screening for low BMD and osteoporosis by chest CT may represent a safe and effective strategy [43, 44].

This study also has some limitations; first, the generalizability of the results may be limited as it was a single-center retrospective study. Therefore, further confirmation of our findings through future studies is necessary. Second, chest CT was performed on different machine models, the impact of which on the measurement of target values will need to be quantified in the future. Third, this study used manual measurements of muscle area and vertebral attenuation values. Although manual selection of ROIs can provide reliable measurements, automated measurements remain indispensable in opportunistic screening. Finally, larger sample sizes and external validations are required to build more accurate diagnostic models and establish fully automated diagnostics.

## Conclusion

In conclusion, in this study, we demonstrated a strong correlation between vertebral HU value, the PMI on chest CT scans, and BMD, indicating that patients with osteoporosis can be opportunistically screened using vertebral HU value and the PMI on chest CT. The combined diagnostic efficacy of T4 HU value, PMI, sex, age, and BMI was higher than that for a one-factor model. Thus, with the increased use of chest CT in screening for lung diseases in the elderly, the opportunistic screening of patients with osteoporosis using chest CT is an economical and safe strategy. Furthermore, more accurate BMD screening for people at a high risk of osteoporosis can improve the early diagnosis of osteoporosis and prevent fragility fractures.

## Data Availability

Data will be available by contacting Youjia Xu, the corresponding author, at the above address.

## Abbreviations

OR: Odds ratio
CI: Confidence intervals
BMD: Bone mineral density
CT: Computed tomography values
PMA: Pectoralis muscle area
PMI: Pectoral muscle index
DXA: Dual-energy X-ray
AUC: Areas under the receiver
OP: Osteoporosis
BMI: Body mass index
ROI: Region of interest
